# Emergency department admissions to the intensive care unit – a national retrospective study

**DOI:** 10.1186/s12873-021-00517-0

**Published:** 2021-10-23

**Authors:** Susanne B. Wilhelms, Daniel B. Wilhelms

**Affiliations:** 1 Department of Anaesthesia and Intensive Care in Linköping, and Department of Biomedical and Clinical Sciences, Linköping University, Linköping, Sweden; 2 Department of Emergency Medicine in Linköping, and Department of Biomedical and Clinical Sciences, Linköping University, Linköping, Sweden

**Keywords:** Emergency medicine, Critical care, Epidemiology, Mortality, International classifications of diseases

## Abstract

**Background:**

Emergency departments (EDs) see a rising number of patients, but only a small fraction of ED patients need immediate intensive care. The characteristics of these patients are mostly unknown and there is reason to believe that there are large inter-hospital differences in thresholds for intensive care admissions from the ED. The purpose of this study was to give a nationwide overview of ED admissions directly to intensive care units.

**Methods:**

We used the Swedish Intensive care Registry to identify all patients admitted to intensive care from the ED (January 1, 2013 until June 7, 2018). The primary outcome was discharge diagnosis after intensive care (primary ICU diagnosis code). ICU mortality and” ICU admission due to only observation” were analyzed as secondary outcomes.

**Results:**

110,072 ICU admissions were included, representing 94,546 unique patients. Intoxication, trauma and neurological conditions were the most common causes for intensive care, but large variations according to age, sex and hospital type were seen. Intoxication was the most prevalent diagnosis in young adults (46.8% of admissions in 18–29 years old), whereas infectious diseases predominated in the elderly (17.0% in 65–79 years old). Overall, ICU mortality was 7.2%, but varied substantially with age, sex, type of hospital and medical condition. Cardiac conditions had the highest mortality rates, reaching 32.9%. The mortality was higher in academic centers compared to rural hospitals (9.3% vs 5.0%). It was more common to be admitted to ICU for only observation in rural hospitals than in academic centers (20.1% vs 7.8%). Being admitted to ICU only for observation was most common in patients with intoxication (30.6%).

**Conclusions:**

Overall, intoxication was the most common cause for ICU admission from the ED. However, causes of ED ICU admissions differ substantially according to age, sex and hospital type. Being admitted to the ICU only for observation was most common in intoxicated patients.

**Trial registration:**

Not applicable (no interventions).

**Supplementary Information:**

The online version contains supplementary material available at 10.1186/s12873-021-00517-0.

## Background

ED patients a are a highly diverse group of patients. Typical ED patients suffer from minor injuries or medical conditions of only moderate severity and are usually treated as outpatients, whereas a small fraction of patients suffer from life-threatening conditions [1, 2]. The critically ill ED patients are often admitted to an intensive care unit.

The use of intensive care resources for selected patient groups or diseases, for example sepsis, are well-described [3]. However, studies focusing on the divergent patient population admitted to the ICU directly from the ED are few, especially in a European setting.

A study by Herring et al., based on data from the United States, indicated that an increasing proportion of patients in the ED require intensive care, with 0.9% of ED patients receiving intensive care in 2001 and 1.6% in 2009, respectively [4]. The most common indications for intensive care admission, in the United States, were chest pain, heart failure and pneumonia [5].

In France, the rates of elderly patients (80 years and older) deemed eligible for ICU admission from the ED ranged from 5.6 to 38.8% across 15 participating centers [6], indicating large hospital-dependent differences in ICU admissions. A similar tendency has been reported from the United States, where 3–55% of the hospitalized patients were admitted to intensive care during their hospital stay [7]. Furthermore, a study by Mathews et al. indicated that ICU admission decisions were often affected by medical ICU bed availability [8]. However, intensive care is a highly limited resource, and it is agreed that intensive care should be reserved for selected patients – those who are too sick to be treated at a general ward but who could still benefit from escalated care involving for example organ supporting techniques [9].

The ICU bed capacity, as well as the definition of “intensive care”, may differ significantly between countries [10], and in many countries, such as Sweden, there are also large within-country variation of ICU bed capacity [11]. Most ICUs in Sweden are level 2 or level 3 ICUs, i.e. they can provide invasive monitoring and basic life support for a short period (level 2) or a full spectrum of monitoring and life support technologies and serve as a regional resource for intensive care (level 3). Level 2 ICUs are most common in rural areas, whereas all academic ICUs are level 3 ICUs. These geographical differences may influence indications for ICU admission, and may result in regional differences in which patients are admitted to the ICU directly from the ED.

Furthermore, the interface between emergency medicine and intensive care is a recurring topic for discussion in European healthcare systems where emergency medicine is being implemented as an independent specialty [12]. A key to informing this discussion is to understand the panorama of critically ill patients which emergency physician actually meet on a frequent basis.

Today, however, the characteristics of patients directly admitted to the ICU from the ED are largely unknown. At the same time, familiarity with the basic panorama of critically ill patients typically presenting in the ED is important knowledge for the emergency physician, as well as for planning and management of intensive care resources in a healthcare system as a whole.

## Methods

The aim of this study was to give an overview of intensive care admissions from the ED in a national cohort of Swedish patients who were directly admitted to the ICU from the ED.

We used data from the Swedish intensive care Registry (from January 1, 2013 until June 7, 2018) to identify patients admitted to the ICU directly from the emergency department.

### The Swedish intensive care registry

About 92% of the Swedish ICUs reported data to the SIR in 2013 [13] and the coverage is representative with regards to both ICU type and geography. Data are reported to SIR prospectively during the ICU stay and the registry has an automatic check for logical errors. Further, local validations of diagnoses are done, but the registry has not been generally/externally validated. The registry has been extensively used in previous studies [14–16]. Variables included are, for example, personal identification number, age, gender, admission and discharge date, primary ICU diagnosis, secondary ICU diagnoses, SAPS3 scoring and outcome (dead, alive). SAPS3 is an internationally developed risk adjustment system for critically ill patients, based on patient characteristics before intensive care, indications for intensive care and physiological parameters at ICU admission [17].

All patients in the Swedish intensive care Registry with either reported admission type “from the emergency department” or “emergency department” reported in the SAPS3 scoring were included.

The conditions leading to ICU admission were analyzed by using the intensive care related primary diagnosis codes (International Classification of Diseases (ICD) codes, Swedish version 10), which were registered at discharge from the ICU. After data from the SIR was extracted, all occurring ICD codes were grouped into organ or disease specific categories by the authors to allow for comparisons between groups. These categories were developed taking the ICD system as well as disease pathogenesis in consideration and are described in more detail in an additional file (see Additional file 1). The SIR variable “hospital type” was used to divide data in academic (Swedish: universitetssjukhus), community (Swedish: länssjukhus) and rural (Swedish: länsdelssjukhus) hospitals. The primary outcome analyzed was primary discharge diagnosis after intensive care (primary ICU diagnosis code). ICU mortality and” ICU admission due to only observation” were analyzed as secondary outcomes. To define “ICU admission due to only observation” we used SAPS3, which is an ICU scoring system to predict mortality [17]. In SAPS3, indication for intensive care is one of the variables used to calculate risk for mortality [17]. If the patient was admitted to ICU for “only observation” according to the patient’s SAPS 3 data registered, the patient was regarded as admitted due to only observation.

The study protocol was approved by the Regional Ethics Review Board in Linköping, Sweden (2018/177–31), and the requirement for informed consent was waived by the Regional Ethics Review Board in Linköping. All methods were performed in accordance with the Declaration of Helsinki and relevant Swedish regulations.

### Statistics

All data were analyzed in STATA version 14 (StataCorp, College Station, TX, USA). Demographic data were reported as mean (standard deviation). Groups of patients were compared using the Student’s t-test (continuous data), the chi-square test (categorical data) or ANOVA. Differences were considered statistically significant at a *p*-value of < 0.05. Due to the large sample size and relatively small number of repeats, no adjustments for repeated ICU admissions were done.

## Results

**Fig. 1:**
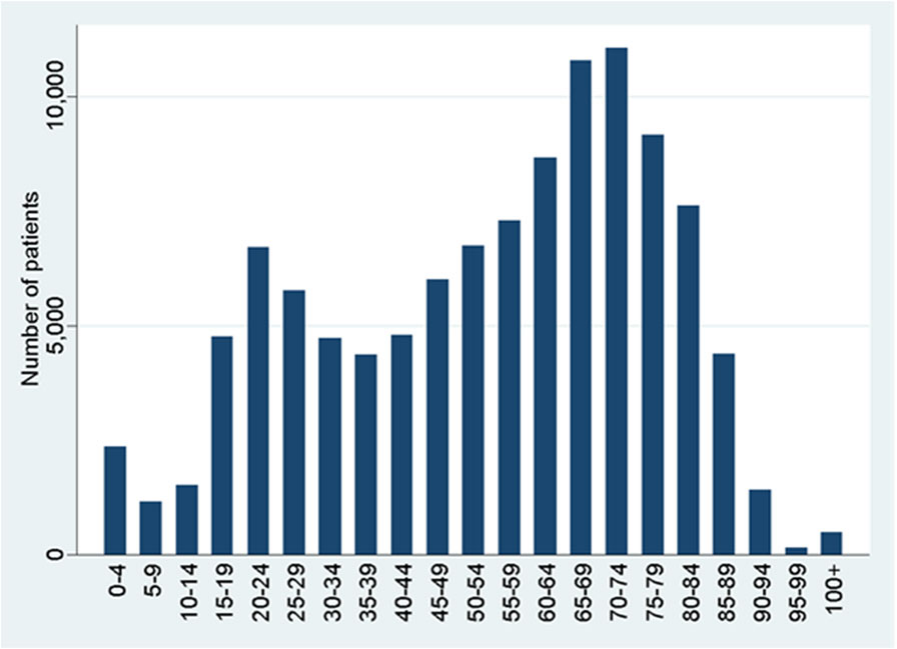
Age characteristics of ICU patients admitted from the emergency department

110,072 ICU admissions were included in the study, including 94,546 unique patients. 4177 patients were admitted more than once to the ICU with the same primary ICU diagnosis. Overall, most of the patients were males (57.7%, *n* = 63,320). The mean age was 53.8 years (451 missing values) with the age intervals 65–69 years and 70–74 years being the most common (Fig. 1). The overall ICU-mortality was 7.2% (*n* = 7887). All admissions in rural and community hospitals and most of the admissions (93.7%) in academic hospitals were admissions to general ICUs (including admissions to neuro critical care and burn critical care). Some of the admissions in academic hospitals were admissions to pediatric intensive care (6.0%) or thoracic critical care (0.1%).

A total of 109,111 admissions had a primary diagnosis registered (*n* = 961 missing). The most common ICU discharge diagnosis for admissions directly from the emergency department was intoxication (19.1% of all admissions, *n* = 20,845). In addition, trauma (14.7%, *n* = 15,981), neurological conditions (12.6%, *n* = 13,706) and infectious diseases (12.2%, *n* = 13,275) were prevalent ICU discharge diagnoses.

### Age

The ICU discharge diagnoses clearly differed between age categories (Table 1). Intoxication was most common in young adults, reaching 46.8% of ICU admissions in patients 18–29 years old. In the elderly, infectious diseases were the most common discharge diagnoses (17.0% in patients 65–79 years old, 16.8% in patients 80 years and older). Young children were commonly admitted to ICU with a neurological disease or symptom (26.2% in patients 0–1 years old, 30.7% in patients 2–9 years old). On the other hand, intoxication was uncommon in both young children and in the elderly.

**Table 1:** The 10 most common causes of ICU admission related to age (%)

	overall	0–1 y	2–9 y	10–17 y	18–29 y	30–44 y	45–64 y	65–79 y	80+ y	
	(*n* = 109,111)	(*n* = 1380)	(*n* = 2137)	(*n* = 3833)	(*n* = 14,833)	(*n* = 13,819)	(*n* = 28,509)	(*n* = 30,702)	(*n* = 13,898)	**Most prevalent ICD code**
**intoxication**	19.1	3.9	3.5	33.2	46.8	39.4	18.6	4.0	3.7	F100 Acute alcohol intoxication
**trauma**	14.7	14.5	26.6	27.0	18.7	16.3	14.3	10.8	12.8	T079 Unspecified multiple injuries
**neurology**	12.6	26.2	30.7	8.8	6.4	9.7	14.8	14.0	11.3	R568 Unspecified seizures
**infection**	12.2	19.5	9.2	4.5	4.3	7.8	11.8	17.0	16.8	R572 Septic chock
**respiratory**	8.5	12.4	6.9	3.4	3.3	3.8	6.9	13.4	12.5	J969 Unspecified respiratory dysfunction
**cardiology**	8.4	6.2	3.2	2.3	2.1	3.3	7.8	12.8	14.1	I469 Unspecified cardiac arrest
**endocrinology**	8.0	6.3	9.9	11.6	8.2	6.5	8.3	8.0	7.5	E141 Unspecified diabetes with ketoacidosis
**gastrointestinal**	5.5	1.2	1.5	1.1	1.0	2.7	6.5	7.3	9.3	K922 Unspecified gastrointestinal bleeding
**circulatory**	2.9	0.7	0.4	0.7	0.5	1.0	2.7	4.7	4.7	I710 Aortic dissection
**consciousness**	2.8	1.2	2.2	3.2	3.7	4.0	2.8	2.3	2.3	R402 Unspecified coma

#### Sex

Overall, intoxication was the most common ICU discharge diagnosis in females (22.4%, *n* = 10,371), whereas trauma was most common in males (18.1%, *n* = 11,356) (Table 2). Intoxication was also one of the most common reasons for ICU admission in males, reaching 16.7% (*n* = 10,474) of all (male) ICU admissions directly from the ED. On the other hand, traumatic injuries in need for intensive care were significantly fewer in females (10.0%, *n* = 4625) compared to males.

**Table 2:** The ten most common reasons for ICU admission directly from the emergency department (%), related to sex

	overall	male	female	p
	(*n* = 109,111)	(*n* = 62,730)	(*n* = 46,381)	
**intoxication**	19.1	16.7	22.4	< 0.01
**trauma**	14.7	18.1	10.0	< 0.01
**neurology**	12.6	12.2	13.0	< 0.01
**infection**	12.2	12.3	12.0	0.25
**respiratory**	8.5	7.5	9.8	< 0.01
**cardiology**	8.4	9.1	7.4	< 0.01
**endocrinology**	8.0	7.0	9.2	< 0.01
**gastrointestinal**	5.5	5.7	5.1	< 0.01
**circulatory**	2.9	3.1	2.6	< 0.01
**consciousness**	2.8	2.8	2.9	0.65

#### Hospital type

The ICU-related primary diagnoses differed between hospital types (Table 3). Intoxication was the most common ICU discharge diagnosis in rural hospitals (22.9%, *n* = 7773), whereas intoxication-related diagnoses were more uncommon in academic centers (12.8%, *n* = 3099). On the other hand, diagnoses related to trauma, neurological, and cardiac conditions were more common in academic centers compared to other types of hospitals. Furthermore, the SAPS3 value was higher in academic centers (mean 54.9 ± 16.0) than in community hospitals (52.1 ± 15.7) and rural hospitals (49.6 ± 14.5) (*p* < 0.01).

**Table 3:** The ten most common reasons for ICU admission directly from the emergency department (%), related to type of hospital

	Academic	Community	Rural	p
	(*n* = 24,180)	(*n* = 50,972)	(*n* = 33,959)	
**intoxication**	12.8	19.6	22.9	< 0.01
**trauma**	17.5	14.1	13.4	< 0.01
**neurology**	15.1	11.8	11.9	< 0.01
**infection**	11.9	11.9	12.8	0.02
**respiratory**	8.5	8.6	8.4	0.10
**cardiology**	11.6	8.2	6.3	< 0.01
**endocrinology**	6.6	9.0	7.4	< 0.01
**gastrointestinal**	4.0	5.4	6.6	< 0.01
**circulatory**	3.1	3.0	2.4	< 0.01
**consciousness**	3.1	3.1	2.3	< 0.01

#### SAPS3-related cause of admission

The most common cause of ICU admission according to SAPS3 registration was a neurological cause, which entails decreased level of consciousness and seizures, followed by respiratory and cardiovascular dysfunction (see Supplementary Table 1).

### ICU admission for only observation

A minority of the ICU admissions, 12.5%, was primarily for observation. This was most common in patients with intoxications (30.6% of the admissions). ICU admission for observation was also more common in rural hospitals (20.1%), compared to academic and community hospitals (7.8 and 9.6%, respectively).

### ICU mortality

The mortality rates were similar in males and females (7.2 and 7.1%, respectively), and the mortality rates increased with age (Table 4). The mortality rates were also higher in academic hospitals (9.3%) than in community hospitals (7.6%) and rural hospitals (5.0%).

**Table 4:** Mortality (%) according to the ten most common causes of ICU admission

	Overall	Male	Female	Academic	Community	Rural	0–1 y	2–9 y	10–17 y	18–29 y	30–44 y	45–64 y	65–79 y	80+ y
	**(*n*** **= 110,072)**	**(*n*** **= 63,320)**	**(*n*** **= 46,752)**	**(*n*** **= 24,365)**	**(*n*** **= 50,972)**	**(*n*** **= 34,735)**	**(*n*** **= 1381)**	**(*n*** **= 2144)**	**(*n*** **= 3846)**	**(*n*** **= 14,923)**	**(*n*** **= 13,919)**	**(*n*** **= 28,739)**	**(*n*** **= 31,037)**	**(*n*** **= 14,083)**
**all admissions**	7.2	7.2	7.1	9.3	7.6	5.0	1.6	1.5	1.3	1.5	2.7	6.2	11.3	13.5
**intoxication**	0.27	0.29	0.26	0.32	0.3	0.2	0.0	0.0	0.0	0.1	0.11	0.36	1.0	2.5
**trauma**	4.5	4.2	5.2	6.1	4.5	2.8	1.0	1.2	1.5	1.2	2.0	3.2	8.1	12.1
**neurology**	8.4	7.9	9.0	10.3	9.1	5.5	0.0	0.0	1.2	2.6	5.0	7.8	12.1	13.2
**infection**	9.9	9.3	10.8	9.6	11.4	8.0	0.0	2.6	2.3	1.7	3.5	8.1	12.2	15.0
**respiratory**	6.3	6.2	6.4	4.8	6.5	7.1	0.6	2.0	0.8	1.2	3.8	4.1	7.0	10.7
**cardiology**	32.9	32.6	33.4	35.4	35.4	24.5	18.8	18.8	23.9	40.3	39.0	31.3	33.6	32.0
**endocrinology**	1.2	1.2	1.2	1.2	1.0	1.5	0.0	1.0	0.0	0.1	0.3	1.2	1.8	2.5
**gastrointestinal**	6.4	5.6	7.4	8.9	6.9	4.5	5.9	3.0	4.9	2.7	2.5	6.2	6.6	7.9
**circulatory**	10.6	10.6	10.5	9.6	11.2	10.4	11.1	11.1	0.0	1.3	1.5	7.5	11.0	16.9
**consciousness**	2.6	2.3	3.0	1.9	2.9	2.6	0.0	0.0	0.0	0.0	0.9	2.6	5.2	5.7

Patients admitted to the ICU because of cardiac conditions had a remarkably high ICU mortality of 32.9%. Other conditions with high mortality, over 10%, were circulatory diseases, cancer and rheumatic disorders (few observations). On the other hand, the most common cause of admission, intoxication, had a low overall mortality of 0.27%.

## Discussion

The causes of ICU admission directly from the ED clearly varied with both age, sex and type of hospital.

Overall, the finding that intoxication was such a common reason for ICU admission in the adult population stands out. Intoxication caused 46.8% of all ICU admissions in young adults (18–29 years) and 39.4% of the admissions in the age group 30–44 years, which means that a large share of all ICU resources on a national level in Sweden are used to care for intoxicated patients. Yet, overall mortality in intoxicated patients was less than 1 %. Due to lack of data, we could not distinguish between intentional and non-intentional intoxications in the study cohort. According to previous studies on poisoning in adolescents and adults it is, however, reasonable to assume that most of the intoxications were intentional [18, 19].

In the elderly, infectious diseases were the most common reason for ICU admission. This finding stands in contrast to previous European studies, most notably Fassier et al., who reported that respiratory-related diagnoses, and especially acute pulmonary edema, were the most common causes of ICU admission in elderly (≥80 years) patients in France [20]. In addition, in a study by Flaatten et al., respiratory failure was reported as the most common cause of ICU admission in patients ≥80 years old [21]. There may be several reasons for these discrepancies. In the study by Fassier et al., pneumonia was defined as a respiratory condition whereas, in this study, we defined it as an infectious disease. Further, another reason for the differences may be that the studies by Fassier et al. and Flaatten et al. also included patients admitted to the ICU from a ward, not only from the ED, which is likely to alter the spectrum of underlying medical conditions.

In children, the most common causes for intensive care admission from the emergency department were neurological conditions (< 9 years) and intoxications (10–17 years). This finding also differs from a previous study in England and Wales, where respiratory and cardiovascular causes were more common [22]. However, like most earlier studies in the adult population, this study also included patients admitted to ICU from a ward. Other explanations for the discrepancies may be different organization of the health care systems in Sweden and the UK.

The most common cause of ICU admission in males was trauma. According to a previous study from another Scandinavian country, Finland, trauma resulting in intensive care was similarly more common in males than in females [23].

In contrast, intoxication was the most common cause of intensive care admission in females. In fact, the absolute number of males and females receiving intensive care because of intoxication was almost similar, but due to fewer female ICU patients overall, the percentages differ (22.4% of females; 16.7% of males). This result is in line with an earlier study by Lindqvist et al., where ICU-treated intoxication was as common in males as in females [24].

In our study, the panorama of causes for ICU admission as well as the mortality rates differed between hospital types. One reason for this may be that some advanced care is centralized to the academic centers in Sweden [25]. However, this is unlikely to be the sole explanation, since we only included patients who were directly admitted to the ICU from the ED and patients usually visit the ED which is geographically closest to their home. Another probable reason for regional differences could be differences in hospital organization. Larger hospitals often provide intermediate care units for patients in need of continuous monitoring of vital signs but without need for advanced intensive care. In hospitals without intermediate care facilities, often rural hospitals, these patients are typically admitted to the ICU instead. This model of explanation may also be supported by the fact that ICU patients in academic hospitals seem to be sicker than in rural hospitals, as indicated by the SAPS3 value at admission as well as a higher mortality in the ICU.

Deciding on the right level of care for the individual patient is one of the main challenges in the ED. In times of ICU bed shortage, it is even more important to admit the “right” patients to the ICU; those who are too sick to be admitted to a general ward but who could still benefit from ICU care. The COVID-19 pandemic, with a further aggravated shortage of ICU beds, has highlighted the need to optimize ICU bed utilization [26]. However, even in non-pandemic times, ICU bed capacity varies over time and shortages are prevalent in many hospitals [27, 28]. Generally, Sweden has a low ICU bed capacity per capita, compared to other developed countries [29]. Thus, optimization of ICU bed utilization is a constant challenge and identification of ICU patient who would potentially be managed safely at a lower level of care is one potential avenue to reduce ICU strain.

In our study, 12.5% of the patients were admitted to the ICU for observation. This was most common in intoxicated patients, of whom one third were admitted to an ICU mainly for observation. Further, intoxicated patients had exceptionally low mortality (0.3%) compared to other groups of ICU patients. Taken together, these findings raise the question whether a large share of intoxicated patients admitted to the ICU from the ED really benefit from ICU care, or if they could instead be as safely managed in intermediate care wards or on general wards provided that the staff is trained accordingly, and relevant standard operating procedures are in place. To answer this question, an important first step will be to develop tools to help identify patients with a high risk for adverse events among intoxicated ED patients considered for ICU admission.

The present study, being a retrospective, register-based investigation, has some general limitations. Firstly, coding of diagnoses is done at ICU discharge, which entails a risk of recall bias. However, the coding is typically done by the attending intensive care physician who has been responsible for the individual patient, which should reduce the risk of incorrect coding. Further, the Swedish Intensive Care registry is validated for logical errors, but there is no detailed validation of the coding. There is no linkage between the coding in the Swedish Intensive care Registry and economic compensation, either to the hospital or the individual physician. Thus, it is unlikely that coding is influenced by economic incentives.

Since the present study is based only on Swedish data, we are unable to determine the generalizability of our findings to other countries and healthcare systems. Most likely, the panorama of ICU discharge diagnoses will vary according to the overall panorama of disease in the population, as well as factors associated with the organization of the healthcare system. This warrants for similar studies in other countries.

In summary, the most common ICU discharge codes for patients admitted from the ED were intoxication, trauma and neurological conditions. However, ICU admissions from the ED vary substantially with age, sex and hospital type.

## Supplementary Information

**Additional file 1.**

**Additional file 2.**

## Data Availability

The data that support the findings of this study are available from the Swedish intensive care registry but restrictions apply to the availability of these data, which were used under license for the current study, and so are not publicly available. Data are however available from the authors upon reasonable request and with permission of the Swedish intensive care registry.

## Abbreviations

ED: emergency department
ICD: International Classification of Diseases
ICU: intensive care unit
SAPS3: Simplified Acute Physiology Score-3
