# Supplementary material for: Emergency department admissions to the intensive care unit – a national retrospective study
Source: BMC Emerg Med. 2021 Oct 23;21:122. doi: 10.1186/s12873-021-00517-0 (PMC8540137; doi:10.1186/s12873-021-00517-0)
Supplement: Supplementary file 1 — Additional file 1. [file 12873_2021_517_MOESM1_ESM.docx]

**APPENDIX 1:** describe all organ or disease specific categories of ICD codes

**Cancer:** C72.9, C80.9, C96.9, D33.9, D43.9, D48.9

**Cardiology:** I20.9, I21.4, I21.9, I22.9, I24.1, I27.9, I30.9, I31.9, I35.0, I42.9, I44.2, I44.3, I45.9, I46.0, I46.9, I47.2, I47.2A, I47.2B, I48.9, I49.0, I49.5, I49.9, I50.1, I50.9, I51.9, R00.0, R00.1, R07.4, R57.0, Z03.4

**Circulation:** I10.9, I26.0, I26.9, I27.0, I27.8, I71.0, I71.4, I71.8, I71.9, I73.9, I74.3, I74.9, I80.9, I82.9, I95.9, R57.1, R58.9, T67.0

**Complication:** T80.9, T81.0, T81.1, T81.2, T81.3, T81.4, T81.9, T85.0, T85.7, T88.3, T88.4, T88.5, T88.7, T88.9, Y40.9, Y42.3, Y43.3, Y44.2, Y44.5, Y45.0, Y47.9, Y57.5, Y63.9, Y69.9, Y83.2

**Consciousness:** R40.0, R40.2, R41.0, R55.9

**Dermatology:** L08.9, L27.0, L89.9, L98.9

**Endocrinology:** E03.5, E05.5, E10.0A, E10.1, E10.1A, E10.1X, E10.8, E11.1A, E11.1B, E11.1D, E11.8, E14.0, E14.1, E21.3, E22.2, E23.0, E23.2, E27.4, E34.0, E34.9, E43.9, E51.2, E66.9, E80.2, E83.5, E83.5X, E84.9, E86.9, E87.0, E87.1, E87.1B, E87.2, E87.3, E87.5, E87.6, E87.7, E87.8, E88.9, R73.9

**Gastrointenstinal:** I85.0, I85.9, K12.2, K22.3, K25.0, K25.1, K25.4, K27.1, K27.9, K46.0. K46.1, K52.9, K55.0, K55.9, K56.4, K56.7, K62.5, K63.1, K65.0, K72,9, K81.9, K83.0, K85.9, K92.0, K92.2, R10.4, R18.9, R19.8A

**Gynecology:** N93.9

**Hematology:** D59.3, D64.9, D68.3, D68.9, D75.9, D76.1

**Immunology:** D84.9, T78.2, T78.2A, T78.2B, T78.3, T78.4, Z91.0, Z94.9

**Infection:** A04.7, A09.9, A16.2, A32.1, A39.9, A41.4, A41.9, A46.9, A48.0, A48.1, A48.3, A49.9, A86.9, A87.9, B00.4, B20.9, B34.9, B37.7, B54.9, B59.9, B95.0, B95.1, B95.2, B95.3, B95.5, B95.6, B95.7, B96.2, B96.3, B96.5, B96.8, B99.9, G00.1, G00.9, G03.9, G04.9, G04.9A, G06.0, G06.1, I33.0, I38.9, I40.9, J04.0, J04.2, J05.0, J05.1, J06.9, J09.9, J10.0, J10.1, J10.8, J12.1, J12.8, J12.9, J13.9, J15.8, J15.9, J18.0, J18.1, J18.8, J18.9, J20.9, J21.0, J36.9, J39.0, J95.8A, M72.6, M86.9, R57.2, R65.1, T79.3, U82.2

**Intoxication:** F10.0, F11.0, F12.0, F13.0, F14.0, F15.0, F19.0, R78.0. T39.0, T39.1, T39.3, T40.1, T40.2, T40.6, T40.7, T41.4, T42.3, T42.4, T42.7, T43.0, T43.2, T43.3, T43.5, T43.6, T43.8, T43.9, T44.5, T44.7, T45.5, T46.0, T46.1, T50.9, T51.1, T51.2, T52.3, T52.9, T54.9, T57.3, T58.9, T59.9, T63.0, T63.4, T65.9, Z03.6

**Neonatal:** P27.1, P28.4, P28.8, P59.9, P70.4, P77.9, P90.9B, Q07.9, Q20.9, Q21.0, Q21.3, Q22.0, Q22.3, Q23.4, Q24.5, Q24.9, Q25.1, Q25.4, Q30.0, Q34.9, Q40.0, Q45.9, Q64.9, Q79.0, Q89.9, Q99.9

**Neurology:** F80.3, G12.2, G12.9, G35.9, G40.2C, G40.2X, G40.3, G40.3F, G40.4X, G40.9, G41.0, G41.2, G43.3, G61.0, G70.0, G71.9, G83.8, G83.9, G91.9, G93.1, G93.5, G93.6, G93.8, G95.2, G95.9, G98.9, I60.2, I60.7, I60.9, I61.0, I61.2, I61.3, I61.4, I61.5, I61.9, I62.0, I62.1, I62.9, I63.3, I63.6, I63.8, I63.9, I64.9, I65.9, I66.0, I66.9, I67.8, I67.9, R25.2, R52.9, R56.0, R56.8, R56.8X

**Observation:** Z03.8, Z04.9

**Obstetrics:** O14.1, O14.9, O15.9, O72.3, O88.2, O99.4, O99.5

**Psychiatry:** F05.9, F10.2, F10.2A, F10.4, F13.1, F13.2, F16.1, F19.2, F29.9, F32.9, X84.99

**Renal:** N10.9, N17.8, N17.9, N18.9, N25.1, N39.0

**Respiratory:** G47.3, J38.4, J39.8, J44.0, J44.1, J44.9, J45.1A, J45.9, J46.9, J69.0, J80.9, J80.9A, J80.9B, J08.9C, J80.9X, J81.9, J84.1, J86.9, J93.9, J94.8, J94.9, J95.0, J95.1, J95.2, J96.0, J96.01, J96.9, J96.90, J96.91, J96.99, J98.1, J98.5, R04.9, R06.1, R09.0, R09.2

**Rheumatology:** M00.9, M35.9

**Side effects to medication:** E16.0

**Trauma:** S01.9, S02.1, S02.9, S06.0, S06.1, S06.2, S06.3, S06.4, S06.5, S06.50, S06.6, S06.8, S06.9, S06.90, S12.9, S14.1, S15.9, S17.0, S22.0, S22.4, S24.1, S25.0, S25.9, S26.9, S27.0, S27.00, S27.01, S27.1, S27.3, S27.9, S31.1, S32.8, S34.1, S35.9, S36.0, S36.00, S36.1, S36.9, S37.9, S39.9, S72.0, T04.9, T07.9, T10.9, T11.9, T12.9, T13.9, T14.1A, T14.1B, T14.9, T17.9, T18.1, T18.9, T21.2, T27.3, T27.7, T30.0, T30.4, T35.7, T68.9, T70.2, T70.3, T71.9, T74.9, T75.0, T75.1, T75.4, T79.2, T79.4, T79.6, T79.7, Z04.2, Z04.5
