# Supplementary material for: Emergency department admissions to the intensive care unit – a national retrospective study
Source: BMC Emerg Med. 2021 Oct 23;21:122. doi: 10.1186/s12873-021-00517-0 (PMC8540137; doi:10.1186/s12873-021-00517-0)
Supplement: Supplementary file 2 — Additional file 2. [file 12873_2021_517_MOESM2_ESM.pdf]

| | Only observation? (n=109 111) | SAPS3 cause of ICU admission (n=94 652) (%) | | | | | | | | | |
|---|---|---|---|---|---|---|---|---|---|---|---|
| **Category** | **Observation = yes (%)** | **Cardiovascular** | **Liver** | **Gastrointestinal** | **Neurological** | **Renal** | **Respiratory** | **Hematological** | **Metabolic** | **Trauma** | **Other** |
| Intoxication | 30.6 | 6.83 | 2.3 | 1.3 | 74.8 | 2.3 | 18.3 | 0.5 | 35.3 | 3.0 | 25.7 |
| Trauma | 10.6 | 10.2 | 3.0 | 3.8 | 44.8 | 3.5 | 21.0 | 2.4 | 7.3 | 82.7 | 8.7 |
| Neurology | 7.6 | 6.9 | 0.7 | 0.8 | 98.4 | 2.2 | 13.0 | 1.4 | 7.4 | 3.4 | 5.0 |
| Infection | 6.0 | 57.8 | 5.7 | 11.3 | 26.0 | 29.0 | 52.7 | 6.3 | 27.5 | 1.0 | 12.9 |
| Respiratory | 6.3 | 17.6 | 1.3 | 3.2 | 23.5 | 7.9 | 97.0 | 1.4 | 21.4 | 2.0 | 7.1 |
| Cardiology | 2.8 | 94.5 | 2.9 | 3.1 | 44.0 | 12.6 | 43.6 | 2.0 | 26.7 | 4.0 | 5.3 |
| Endocrinology | 4.9 | 11.0 | 1.9 | 6.8 | 22.8 | 14.7 | 7.1 | 1.0 | 96.8 | 1.4 | 7.4 |
| Gastrointestinal | 7.6 | 37.8 | 17.0 | 84.6 | 10.4 | 15.0 | 12.0 | 9.6 | 18.2 | 1.0 | 6.2 |
| Circulatory | 14.4 | 77.5 | 2.9 | 12.4 | 11.9 | 14.0 | 25.7 | 8.0 | 13.6 | 3.7 | 16.1 |
| Consciousness | 11.4 | 10.5 | 3.4 | 2.4 | 96.4 | 6.6 | 22.8 | 1.1 | 20.1 | 7.4 | 12.2 |
| **Type of hospital** | | | | | | | | | | | |
| Academic | 7.8 | 29.8 | 2.9 | 6.7 | 49.1 | 9.6 | 31.8 | 2.4 | 19.0 | 18.2 | 10.5 |
| Community | 9.6 | 29.0 | 3.7 | 9.5 | 50.3 | 12.0 | 32.7 | 3.0 | 33.2 | 14.2 | 10.6 |
| Rural | 20.1 | 26.9 | 3.6 | 11.1 | 42.5 | 11.4 | 28.8 | 3.3 | 23.9 | 13.3 | 13.7 |
| All | 12.5 | 28.6 | 3.5 | 9.3 | 47.8 | 11.3 | 31.4 | 3.0 | 27.3 | 14.9 | 11.5 |

(A patient can have more than one cause of ICU admission)

**Supplementary table 1:**
describe SAPS3-related causes of ICU admissions
